# Artificial Dentures

**Published:** 1869-03

**Authors:** John Allen


DENTAL REGISTER.

VOL. XXIII.] MARCH, 1869. [No. 3.

### Original Communications.

### ARTIFICIAL DENTURES.

BY JOHN ALLEN, D. D. S.

Notwithstanding all that has been done to arrest and prevent the decay and loss of the human teeth in this country, the work of destruction still goes on, and millions of these important organs are lost by the people of the United States every year.

And yet it is generally conceded, that America has better Dentists, and more of them in proportion to population, than any other nation on the globe; and they are doing all in their power to stay the progress of this national calamity with which we are afflicted, and their timely aid has been crowned with unparalleled success. But still, the immense number of teeth that are annually lost causes a great demand for artificial dentures, which constitutes a large and important branch of Dental practice.

In the construction of these substitutes we should approximate as nearly as possible to the natural organs, keeping the mind's eye upon at least three important points to be attained, viz.: mastication, enunciation, and restoration of the natural form and expression of the teeth, mouth, and face. But how to attain these ends under all the different circumstances we meet with in this department, is a problem not so easily solved as many suppose; for artificial Dentistry differs widely from any other branch of business pertaining to mechanism.

For example: The mechanic works by well known rules and laws, that have been long and well established; and he follows the same routine with his rule, compass and square, that thousands of others did who preceded him, and all producing the same practical results. The architect of the present day has the same well established principles to guide him now, that were employed by the ancients two thousand years ago.

The different styles of architecture known as Doric, Ionic, and Corinthian, were the favorite orders among the Greeks and Romans in their most palmy days, and these orders, with slight modifications, have been transmitted with mathematical precision to the present time.

Watchmaking is all done by fixed rules, which the workmen have only to follow in order to produce good timepieces. Thousands of those little wheels are made just alike, and placed in cases in precisely the same relative position to each other, and all will serve exactly the purposes intended.

Numerous branches of mechanism are successfully pursued by men of moderate capacity, by simply adhering to certain fixed rules and principles in executing their work.

But in the construction of artificial dentures, there are no fixed rules to guide the Dentist, for he has no two cases alike; therefore a rule that would apply in one instance, would not hold good in another. If he should make a thousand sets of teeth, all just alike, upon one model, he would find but one set out of that whole number that could be worn, and that only by the one person from whose mouth the model was taken. Therefore instead of working by rule and scribe, as the mechanic does, the skillful Dentist is ever devising ways and means to meet the various requirements of each particular case. Let us look for a moment at some of the manifold varieties of cases that occur in Dental practice.

For example: One set of teeth must be long, another short; another large, another small. One patient requires prominent teeth, another those that recede; some sets should be irregular, others symmetrical. Mrs. Brown requires dark teeth, Mrs. White, light ones, and Mrs. Jones a shade between.

Miss Prim was extremely well satisfied with herself, until she lost her teeth; and in having them replaced, she must have her own natural, lovely expression again, or she will be unhappy the rest of her life. And if the Dentist fails to restore her former appearance truthfully, he will become the object of her anathematism for years to come. In order to produce a pleasing and natural expression of the teeth, they should be in perfect harmony with the other features of the face. It is not always the most beautiful and symmetrical artificial teeth which appear best in the mouth. On the contrary, slight irregularities often appear the most natural.

The teeth give character to the physiognomy of persons; therefore as great a variety of expressions should be given them as there are individuals for whom they are intended. Here the skill of the artist is required in order to avoid an unnatural contrast, that would lead to detection; for you will recollect, it is the height of art to conceal art.

" The Dentist who is a true artisan, is not ambitious to have his work bear the impress of artificial teeth, but on the contrary, that they should possess that depth of tone, natural form, and truthful expression which characterize the natural organs.

Varying the *position* of the teeth will change the appearance of the mouth, just in proportion as they differ from the natural teeth. Hence, in many persons, their former expression is entirely lost, and distortion has taken the place of symmetry.

A want of taste and skill in the construction and adaptation of artificial teeth, results in rude and graceless work, which contrasts widely with that of the true artisan, who carefully studies the tone, position, and expression of every tooth, and restores the harmony which nature had originally stamped upon the features of his patient.

A few slight touches of the brush in the hands of a skillful artist, will change the whole expression of his picture. So with the teeth; a slight inclination, outward or inward, or variation in length, will change the entire expression of the mouth."

Again, the deflection of the various muscles of the face, consequent upon the loss of the natural teeth, presents another class of physiognomical defects, which also comes within the range of Dental practice; and the time has come, when the Dentist is expected to raise the sunken portions of the face to their original contour by artificial means.

Whether this *could* be done without injury to the muscles thus raised, remained a problem to be solved by an American dentist. This question being settled for all coming time, that no injury results from wearing properly constructed dentures with attachments for this purpose, it has now become a practical and important feature in Dental prosthesis.

The sunken portions of the face can be raised by means of attachments or prominences made upon the denture of such form and size as to meet the requirements of the various cases that are presented to the practitioner.

In view of the facts here presented, and of what is required of the Dentist of the present day, we would urge the importance of a higher standard of qualifications in this department than seems to have been attained by a majority of those who are engaged in this branch of our profession.

These qualifications may be classified as follows:

1st. Surgical, embracing especially all operations pertaining to the preparation of the mouth, and restoring the same to a healthy condition. 2d. Mechanism, or all that which pertains to the manual execution of the work, including impressions, models, dies, plates, mounting of the teeth, etc., etc. 3d. Dental chemistry in general, and especially of the chemical properties of the various substances used for artificial dentures, and the mode of preparing and compounding the different minerals, fluxes, oxides, etc., that are employed in forming Dental substitutes. 4th. Metallurgy, including the different processes of working, alloying, and adapting the different metals used in this branch of our profession. 5th. Anatomy, especially of the bony frame work, and muscles of the head and face, including the different locations, connections, and functions of all the parts which give form and expression to the features of the face. 6th. Artistic qualifications which combine all of the preceding requirements, and constitutes the acme and crowning point of the whole.

As the necessity for these qualifications are self-evident in the construction of artificial dentures, we will dwell only for a moment upon the last two named, as they seem to be practically the least understood by many in our profession.

The face, as you are aware, is formed of different bones and muscles, which give it shape and expression. When the teeth are lost, and a consequent absorption of the alveolar processes takes place, several of these muscles are liable to fall in or become sunken, in a greater or less degree, according to temperament. And, in order to restore them to their former position, the Dentist should be familiar with the form and position of every bone of the face, and know the origin and insertion of every muscle, and what ones to raise, and where to apply attachments to the denture; otherwise, he may produce distortion instead of restoration, by underlaying other muscles than those intended to be raised. Here again, the artistic skill of the Dentist is brought into requisition. He should study the face of his patient as the artist studies his picture, for he displays his talents not upon canvas, but upon the living features of the face; and of how much more importance is the living picture which reflects even the emotions of the heart, than the lifeless form upon canvas. In raising the different muscles of the face, the true artist will carefully avoid producing a stiff, restrained, or puffed appearance. He will place the prominences upon the dentures in their proper position, and make them of such form and size as to allow the muscles to rest, move or play upon them, with perfect ease; that they may again reflect those sensitive emotions which tell of the inner workings of the mind. Or, to use the language of Shakspeare,—

"Your face, my thane, is as a book where men may read strange matters."

Another important consideration in the construction of artificial dentures is, that the materials of which they are formed, should be incorrodible or chemically pure.

The importance of a sweet and healthy mouth will be readily perceived, in view of the fact that the food which is taken into the system is moistened with the saliva, and if this becomes vitiated, either from an unhealthy condition of the salivary glands, or by contact with filthy dentures, it exerts a baneful influence on the stomach and alimentary canal, and impairs the general health in a greater or less degree.

This purity of materials we have in the continuous gum work, when properly made, as none of the materials used are corrodible in the slightest degree in the mouth. Again, all the essential points here referred to, can be attained by this mode of constructing artificial dentures. But too much reliance should not be placed upon the mode, for however perfect this may be in itself, artistic taste, skill and judgment are necessary to direct the operator in his manipulations.

Two artists (so called,) may employ the same method, use the same paints, brushes, canvas, etc., in painting a picture.

One will produce a perfect prototype of nature, with all the delicate shades and tints peculiar to her art; while the other, makes a mere daub that is worthless. The same difference exists among men in various other branches of art and science.

In conclusion, allow me again to urge upon our brethren the great importance of bringing into requisition a much higher order of talent in the artificial branch of our profession than has heretofore been employed by a large number of Dentists, whose ambition prompts them to do the cheapest, not the best work.

This low ambition always has a downward rather than an upward tendency, and will place its votaries where they are sure to be found, upon the lower platform of their profession; they are dead weights upon our fraternity, and do much to retard the progress of Dental science.

But, the aspirant for the highest pinnacle of fame, that he may do the greatest amount of good, moves onward and upward. If he meets with obstacles he surmounts them. If difficulties and discouragements stare him in the face, he overcomes them, keeping his mind's eye steadily fixed upon the goal where he has placed his mark; he believes that what ought to be done in his profession, must be done, and he is going to do it. His restless zeal will not permit him to wait for some predecessor to do the pioneering, he does it himself, let the cost be what it may, or the labor ever so much. Let us then seek for higher qualifications in the artificial branch of our profession, and we will then hear much less of mechanical Dentistry, and more of that which is artistic Dentistry.

